# SMAR1 binds to T(C/G) repeat and inhibits tumor progression by regulating miR-371-373 cluster

**DOI:** 10.1038/srep33779

**Published:** 2016-09-27

**Authors:** Jinumary Mathai, Smriti P. K. Mittal, Aftab Alam, Payal Ranade, Devraj Mogare, Sonal Patel, Smita Saxena, Suvankar Ghorai, Abhijeet P. Kulkarni, Samit Chattopadhyay

**Affiliations:** 1 Chromatin and Disease Biology Lab, National Centre for Cell Science, Savitribai Phule Pune University Campus, Ganeshkhind, Pune-411007, India; 2 Department of Zoology, Savitribai Phule Pune University Campus, Pune 411007, India; 3 Bioinformatics Centre, Savitribai Phule Pune University Campus, Pune 411007, India

## Abstract

Chromatin architecture and dynamics are regulated by various histone and non-histone proteins. The matrix attachment region binding proteins (MARBPs) play a central role in chromatin organization and function through numerous regulatory proteins. In the present study, we demonstrate that nuclear matrix protein SMAR1 orchestrates global gene regulation as determined by massively parallel ChIP-sequencing. The study revealed that SMAR1 binds to T(C/G) repeat and targets genes involved in diverse biological pathways. We observe that SMAR1 binds and targets distinctly different genes based on the availability of p53. Our data suggest that SMAR1 binds and regulates one of the imperative microRNA clusters in cancer and metastasis, miR-371-373. It negatively regulates miR-371-373 transcription as confirmed by SMAR1 overexpression and knockdown studies. Further, deletion studies indicate that a ~200 bp region in the miR-371-373 promoter is necessary for SMAR1 binding and transcriptional repression. Recruitment of HDAC1/mSin3A complex by SMAR1, concomitant with alteration of histone marks results in downregulation of the miRNA cluster. The regulation of miR-371-373 by SMAR1 inhibits breast cancer tumorigenesis and metastasis as determined by *in vivo* experiments. Overall, our study highlights the binding of SMAR1 to T(C/G) repeat and its role in cancer through miR-371-373.

The nuclear matrix is an intricate yet dynamic platform composed of two interacting partners, i.e., nucleic acids and proteins. It not only serves as a hub of vital cellular events such as replication, transcription and transcription coupled alternative splicing; but also provides a niche for DNA damage repair and recombination^1^. Among the different factors involved in compaction and tethering of chromatin to the nuclear matrix, the class of S/MAR binding proteins (MARBPs) play crucial role. DNase I hypersensitive sites, known as S/MARs (Scaffold/Matrix Attachment binding regions), often situated in close proximity to promoters and enhancers are the regions to which these MARBPs bind^2^. They work in a consorted fashion with co-activator or co-repressor complexes at MARs, thereby remodeling the chromatin and regulating gene expression in a tissue and context-dependent manner.

SMAR1 (Scaffold/Matrix Attachment region 1), one such MARBP, identified from double positive mouse thymocytes, is reported principally to be a transcriptional regulator. Subsequently, SMAR1 is known to interact with p53 and act as tumor suppressor resulting in tumor regression *in vivo*^3^. SMAR1 inhibits CyclinD1 transcription by recruiting the mSin3A/HDAC1 repressor complex to its promoter^4^. Interestingly, it has been observed that as the grades of breast carcinoma progress, the levels of SMAR1 reduce significantly^5^. It also plays a decisive role in the fate of a cell, in order to decide between cell cycle arrest and apoptosis, by modulating its interaction with p53^6^. Therefore, investigating SMAR1 target genes (both protein coding and non-coding) in a high throughput manner and its dependency on tumor suppressor protein p53 was a lucrative objective that has been addressed in the present work. Massively parallel high throughput ChIP sequencing for SMAR1 was carried out in HCT116 p53^+/+^ and HCT116 p53^−/−^ cell lines to address this objective. A repeat sequence was identified and validated as the binding site of SMAR1. The predicted SMAR1 targets, including microRNAs, were validated and categorized into different biological pathways.

MicroRNAs (miRNAs) are endogenous, evolutionarily conserved, small noncoding RNAs that control post-transcriptional gene regulation^7^. Conventionally they bind to the 3′UTR of their target mRNAs through an imperfect match, thereby suppressing their translation and stability^8^; however, reports suggest that miRs can bind to gene promoters enhancing gene transcription^9^. As single miRNA can regulate a large number of target genes which lie across diverse biological pathways, miRNAs can dictate and control multiple biological processes, including pluripotency, apoptosis, differentiation, development, and diseases including cancer^10^,^11^. MicroRNAs can either act as tumor suppressors, viz., let-7, miR-16-1 and miR-15a or behave like oncogenes (oncomiRs), for example miR-17~92, miR-155, miR-372 and miR-373^12^. Although the mechanisms by which miRNAs regulate the expression of their targets are well documented, factors regulating genesis of miRNAs remain largely unaddressed. Interestingly, many miRNA gene(s) occur in clusters, indicating that they might be regulated by common transcription factors in an all or none fashion. Various MAR binding proteins in association with other transcription factors have been shown to regulate gene clusters at large distance through locus control regions (LCRs)^13^,^14^. Hence we sought to understand the transcriptional regulation of microRNA target genes by SMAR1. Preliminary studies from our lab have suggested that SMAR1 can potentially regulate the expression of numerous microRNAs vital for different physiological processes. MicroRNA-microarray analysis upon SMAR1 over expression and knockdown in HCT116 p53^+/+^ cells identified microRNAs essential for erythropoiesis, pluripotency of stem cells, apoptosis, cell cycle and tumorigenesis (Supplementary Fig. S1). One such interesting cluster of miRNAs important in stem cell pluripotency and cancer progression is the miR-371-373 cluster. In this study we have aimed to establish the transcriptional regulation of this microRNA cluster by the nuclear matrix protein SMAR1 in the context of breast cancer tumorigenesis and metastasis. Thus, we have focused on the role of SMAR1 as a global gene regulator involved in multiple biological pathways, and as a tumor suppressor protein via transcriptional repression of the miR-371-373 cluster.

## Results

### Mapping the genome-wide distribution of the nuclear matrix protein SMAR1

As SMAR1 can alter the function of p53 in response to the severity of DNA damage and differentially drive the cell towards cell cycle arrest or apoptosis^6^, its role as a critical transcriptional switch becomes undisputed. Thus, the dynamic interactions between p53 and SMAR1 can vary the outcome of gene expression significantly and thereby the fate of the cell. Therefore, in order to identify genome-wide targets of SMAR1, chromatin immunoprecipitation combined with massively parallel sequencing (ChIP-Seq) was performed for the nuclear matrix protein SMAR1 using chromatin prepared from HCT116 p53^+/+^ and HCT116 p53^−/−^ pull down samples. The α-SMAR1 antibody used for ChIP was first validated using immunoblotting assays and the ChIP DNA samples prepared using α-SMAR1 antibody were analyzed for presence of known SMAR1 target gene sequence like Cyclin D1 (Supplementary Fig. S2). After such confirmation, the DNA samples were subjected to high-throughput sequencing and the sequence data obtained was subjected to the ChIP-seq data processing pipeline using *in silico* tools. The bowtie alignment statistics is shown in Supplementary Table S3. The data discussed in this publication (raw and processed files) have been submitted to National Center for Biotechnology Information- Gene Expression Omnibus (NCBI-GEO) and are accessible through GEO Series accession number GSE70058 (https://www.ncbi.nlm.nih.gov/geo/query/acc.cgi?acc=GSE70058). The SMAR1 peak coordinates and their gene annotations for both datasets have been provided in Supplementary spreadsheet 1. Detailed visualization of individual peak region for few genes has been shown in Supplementary Fig. S3.

### *In silico* comparison of SMAR1 binding peaks in HCT116 p53^+/+^ and HCT116 p53^−/−^ data sets

**Figure 1:**
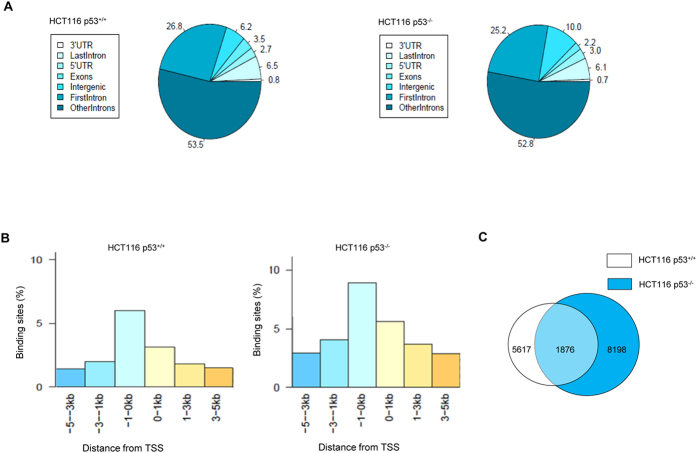
Mapping genome-wide distribution of the nuclear matrix protein SMAR1. (**A)** Distribution of SMAR1-binding sites within specific gene components as analyzed by the Nearest Downstream Gene (NDG) function of PeakAnalyzer software in HCT116 p53^+/+^ and HCT116 p53^−/−^cell lines. (**B)** The percentage of SMAR1 bound peaks within ±5 kb of Transcription Start Site (TSS) for all gene targets in HCT116 p53^+/+^ and HCT116 p53^−/−^cell lines. (**C**) Comparison of SMAR1 gene targets from HCT116 p53^+/+^ and HCT116 p53^−/−^ data sets.

SMAR1 functions by recruiting co-repressor complex to gene promoter or gene body for regulation of transcription, transcription-coupled splicing, DNA damage repair and other vital cellular functions. Hence, we planned to determine the genome-wide binding pattern of SMAR1 and correlate it with transcription. The analysis suggested that SMAR1 has a diverse binding pattern with respect to different gene components. Approximately, 29% of SMAR1 binding was observed in the promoter regions and regulatory elements (5′UTR and first introns) of the gene in both the data sets. Around 53% of SMAR1 binding was detected within the gene body irrespective of the data set (Fig. 1A).

Further, in order to ascertain the binding pattern of SMAR1 influencing nearest gene, the fold enrichment of SMAR1 peaks within −5 and +5 kb of TSS was determined. The genes having SMAR1 peaks within −5 to 0 kb of their TSSs are henceforth termed as downstream target genes. The results revealed that SMAR1 has the highest predisposition to bind upstream of the TSS, i.e., within −1 to 0 kb, which represents the promoter region of the gene. The percent binding of SMAR1 in the promoter region was ~6% in HCT116 p53^+/+^ data set, while ~9% in the HCT116 p53^−/−^ data set (Fig. 1B). The SMAR1 binding was observed to decrease as the distance from the TSS increases. Thus, we found that SMAR1 binds to several components of gene across the chromosomes, but has an enriched occupancy at the gene promoters.

### SMAR1 binds distinct genes depending on p53 status

As SMAR1 and p53 are known to interact and associate with each other, they might regulate downstream target genes either independently or in a co-operative fashion. In order to identify novel target genes regulated by SMAR1 in presence and absence of p53, the data sets for HCT116 p53^+/+^ and HCT116 p53^−/−^ were analyzed and compared for common gene targets using in-house custom Perl scripts. This analysis revealed a total of 1876 genes, which are common to both the data sets, indicating that these gene regions are occupied by SMAR1 irrespective of the p53 status. Also, it was observed that 5617 SMAR1 gene targets were unique to the HCT116 p53^+/+^ data set, signifying their regulation might be dependent on the p53 status. Furthermore, about 8198 gene targets were found to be bound by SMAR1 only in absence of p53, suggesting p53 independent regulation of these genes by SMAR1 (Fig. 1C). Thus, the SMAR1 gene targets can be categorized on the basis of this analysis into three groups: p53 wild type unique (5617 genes), p53 null unique (8198) and common genes (1876). Hence, we conclude that SMAR1 binds and might regulate distinct subsets of genes depending on the availability of p53.

### SMAR1 binds to a T(C/G) repeat DNA

**Figure 2:**
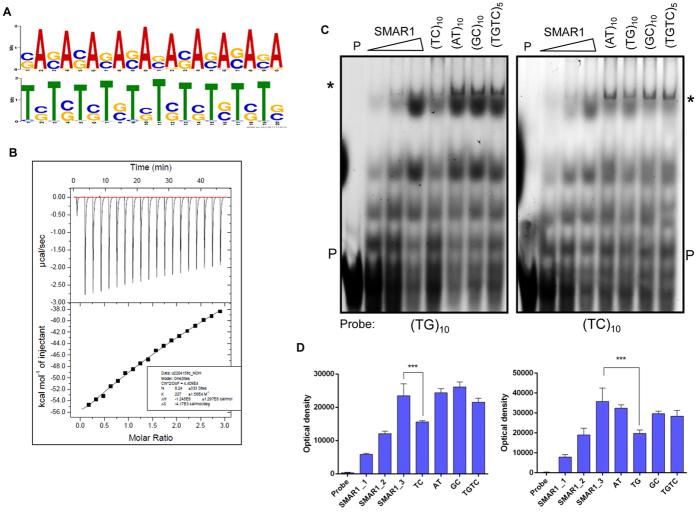
SMAR1 binds to T(C/G) repeat DNA sequence. (**A**) The dinucleotide T(C/G) DNA sequence predicted by the MEME suite as SMAR1 binding motif. (**B)** Isothermal titration calorimetry (ITC) was performed with SMAR1 protein (the macromolecule) and increasing amounts of (TG)_10_ sequence oligo (the ligand). (**C**) Fluorescent electrophoretic mobility shift assay (EMSA) performed using Cy5 tagged motif oligos (TG)_10_ and (TC)_10_ in combination with other competitive non-fluorophore tagged oligos (AT)_10_, (GC)_10_, (TCTG)_5_, (TG)_10_ and (TC)_10_ along with purified recombinant SMAR1 protein and poly (dI-dC). All the gels are run under same experimental condition. (**D**) The densitometry analysis of the supershift bands of interest of Fig. 2C.

In order to determine whether SMAR1 recognizes a particular DNA sequence as its binding site, the sequences corresponding to all peak co-ordinates were retrieved from the UCSC genome browser. The sequences thus obtained were analyzed for the presence of a consensus sequence using MEME-ChIP v4.10.1. The motif search revealed that a dinucleotide T(C/G) repeat sequence was enriched in sequences corresponding to peaks (E-value 1.4e^−2329^) (Fig. 2A). The distribution of length and frequency of occurrence of repeats has been shown in Supplementary Fig. S4. In order to validate the affinity of SMAR1 to this DNA sequence, we carried out an isothermal titration calorimetry to confirm the specificity of interaction between SMAR1 and its T(C/G) DNA binding repeat. The data indicated a linear correlation between the two with increasing amounts of oligo and SMAR1 protein, accompanied with a significant ΔH value (Fig. 2B). We carried out competitive electrophoretic mobility shift assay to further validate this interaction. The results showed a significant increase in SMAR1 binding to both fluorescently tagged oligos in a dose-dependent manner. It was also observed that only (TC)_10_ and (TG)_10_ could compete for binding to SMAR1 protein. The presence of any other untagged, dinucleotide oligos in the reaction mixture did not inhibit the binding of SMAR1 to the fluorophore-tagged oligos (Fig. 2C,D). These results indicate that SMAR1 prefers a dinucleotide T(C/G) DNA repeat sequence for accessing chromatin to exert its regulatory effects.

### SMAR1 binds to gene targets across varied biological pathways

**Figure 3:**
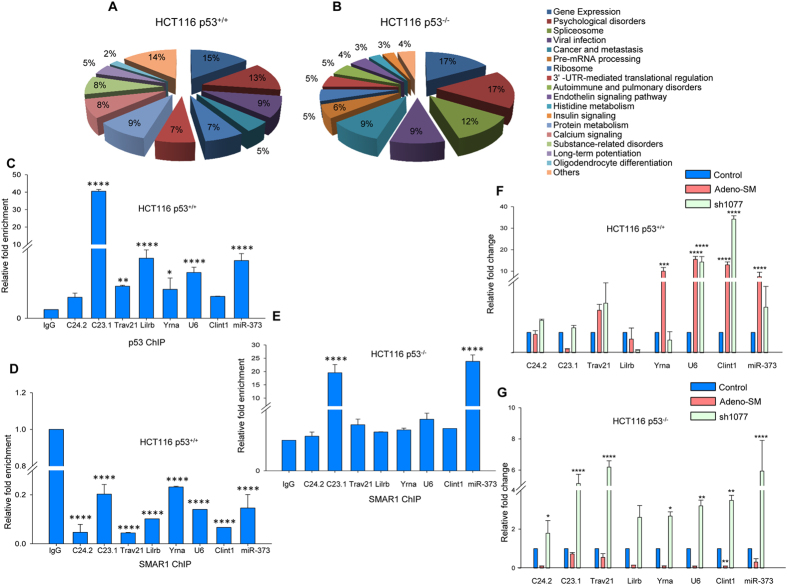
SMAR1 binds and targets distinct genes from various biological pathways depending on p53 status. (**A**) Functional categorization of SMAR1 target genes using DAVID Functional Annotation tool in HCT116 p53^+/+^ data set and (**B**) HCT116 p53^−/−^ data set. (**C**) The gene promoters with p53 binding motif in juxtaposition of SMAR1 binding peaks were amplified in chromatin immunoprecipitate of HCT116 p53^+/+^ by anti-p53 antibody and (**D**) anti-SMAR1 antibody. For convenience, RP11-271C24.2 and RP11-419C23.1 have been labeled as C24.2 and C23.1 respectively. (**E**) The gene promoters with p53 binding motif in juxtaposition of SMAR1 binding peaks were amplified in chromatin immunoprecipitate of HCT116 p53^−/−^ by anti-SMAR1 antibody. (**F**) Quantitative real-time PCR analysis of these gene targets upon overexpression and knockdown of SMAR1 in HCT116 p53^+/+^ cell line. (**G**) Quantitative real-time PCR analysis of these gene targets upon overexpression and knockdown of SMAR1 in HCT116 p53^−/−^ cell line.

As this study was the first of its kind to unravel novel and global gene targets of SMAR1, we were interested in understanding the diverse roles of SMAR1 via control of different downstream target genes. The SMAR1 gene targets in both the data sets as predicted by ChIP-seq analysis were subjected to DAVID Functional Annotation tools^15^. The target genes were then broadly classified into functional categories and selected for critical analysis. The DAVID functional analysis suggested that the SMAR1 gene targets in both the data sets belonged to a broad range of biological processes, viz., splicing, protein and histidine metabolism, pulmonary disorders, viral infection, insulin and calcium signalling, 3′UTR mediated translation regulation, cancer and metastasis, etc. (Fig. 3A,B). A distinct category of microRNA and long non-coding RNA (lncRNA) genes were also predicted to be targeted by SMAR1 (Supplementary Fig. S5).

A number of microRNAs, viz., miR-205, miR-383, miR-572, miR-371-373, miR-100, miR-32, miR-206, miR-147, miR-221/222, etc. playing diverse roles in cancer cell proliferation and metastasis, differentiation, epithelial to mesenchymal transition (EMT), inflammation and many other vital cellular functions have been predicted to be SMAR1 targets by ChIP-seq analysis^16^,^17^,^18^,^19^,^20^,^21^,^22^,^23^,^24^. Thus, our data suggests multi-faceted role of SMAR1 and projects new and unexplored pathways that SMAR1 might orchestrate globally.

### SMAR1 and p53 bind and regulate target genes in a competitive manner

Tumor suppressor p53 is reported to recognize a 10 bp consensus DNA motif sequence 5′-PuPuPuC(A/T)(T/A)GPyPyPy-3′^25^. As SMAR1 is known to associate with p53 and mediate regulation of downstream gene targets^6^,^26^, we were interested to check for the presence of p53 binding site in the vicinity of SMAR1 binding repeat sequence. In order to address this objective, we scanned for the presence of the canonical p53 motif within 50 bp upstream and downstream of peak co-ordinates in the HCT116 p53^+/+^ data set and selected such gene targets for further study^27^. Of the genes that harbour both SMAR1 and p53 binding sites in their promoters, 8 genes (including lncRNAs and miRNA) were selected randomly (Supplementary Table S1) and subjected to chromatin immunoprecipitation in HCT116 p53^+/+^ and HCT116 p53^−/−^ cell lines. The real-time PCR analysis suggested specific binding of p53 in these gene promoters while SMAR1 occupancy was found to be diminished at these regions in HCT116 p53^+/+^ cell line (Fig. 3C,D). However, in absence of p53 in HCT116 p53^−/−^ cell line, SMAR1 was observed to be enriched at these gene promoters (Fig. 3E), suggesting a competitive binding of SMAR1 and p53 at these gene promoters.

Further, we elucidated the effect of SMAR1 occupancy in the regulation of these gene targets, by overexpression and knockdown of SMAR1. As SMAR1 generally mediates gene repression, we wanted to determine the transcript levels of these genes in HCT116 p53^+/+^ and HCT116 p53^−/−^ cell lines. Overexpression and knockdown of SMAR1 in HCT116 p53^+/+^ cells did not correlate inversely with the gene transcript levels (Fig. 3F). In these cells, the genes RP11-271C24.2, RP11-419C23.1 and LILRB did not show significant change either upon SMAR1 overexpression or downregulation. TRAV21, U6, CLINT1 and miR-373 showed elevated levels irrespective of SMAR1 overexpression or knockdown. The Y RNA gene exhibited induced transcription upon Adeno-SM treatment but remains unaltered upon SMAR1 knockdown by sh1077 construct. However, in HCT116 p53^−/−^ cell line, SMAR1 overexpression reduced the gene transcription and knockdown of SMAR1 considerably increased all the gene transcripts as detected by real time PCR (Fig. 3G). As we observed reduced expression levels of miR-373 in HCT116 cell lines, due to its epigenetic silencing^28^, we determined the miR-373 expression across various cancer cell lines (Supplementary Fig. S6). We observed that miR-373 expression was significant in breast cancer lines. Hence, we have used breast cancer as our model system for studying regulation of miR-373. Thus, we conclude that SMAR1 and p53 compete with each other for gene promoter occupancy as well as gene regulation.

### SMAR1 binds and recruits repressor complex upstream of miR-371-373 cluster

**Figure 4:**
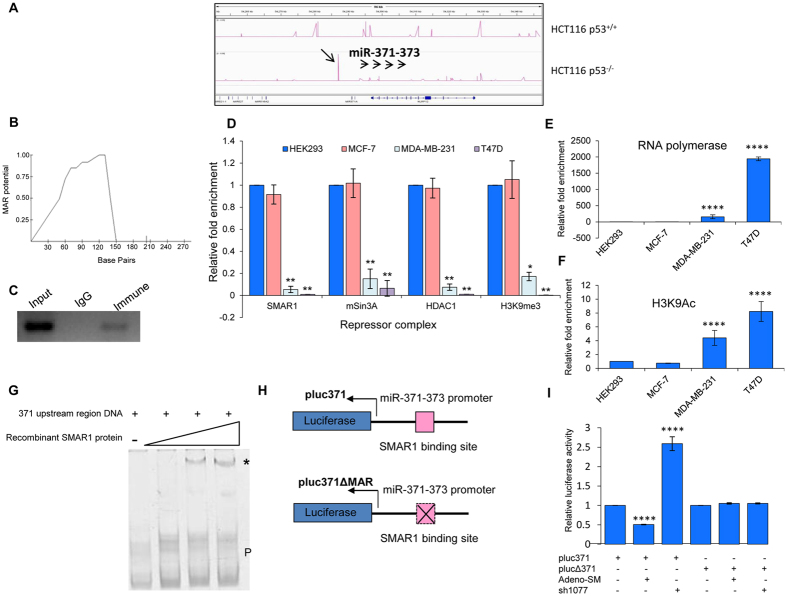
SMAR1 binds and recruits repressor complex upstream of miR-371-373 cluster. (**A**) Peak visualization of ChIP-seq predicted binding of SMAR1 at miR-371-373 promoter in HCT116 p53^+/+^ and HCT116 p53^−/−^ cells. (**B**) MAR-Wiz analysis for the miR-371-373 promoter showing presence of a potential MAR. X-axis denotes base pairs and Y-axis denotes MAR potential. (**C**) Chromatin immunoprecipitation (ChIP) was performed in MCF-7 cells by α-SMAR1 antibody using miR-371-373 promoter specific primers. Rabbit IgG was used as a control. Gel image shown here is the cropped version of whole gel image given in Supplementary Fig. S7. (**D**) ChIP followed by quantitative real time PCR (qRT PCR) was performed for detecting enrichment of SMAR1, mSin3A, HDAC1 and H3K9me3 at the miR-371-373 promoter in four cell lines, HEK293, MCF-7, MDA-MB-231 and T47D. (**E**) ChIP followed by quantitative real time PCR was performed for detecting enrichment of RNA polymerase II at the miR-371-373 promoter in all four cell lines. (**F**) The presence of histone active mark H3K9Ac was detected by ChIP-qRT PCR at the miR-371-373 promoter in all four cell lines. (**G**) Electrophoretic mobility shift assay (EMSA) performed for determining the presence of SMAR1 binding upstream of the miR-371-373 cluster. (**H**) Diagrammatic representation of pluc371 and pluc371ΔMAR constructs. (**I**) Relative luciferase activity of pluc371 and pluc371ΔMAR constructs upon ectopic expression and knockdown of SMAR1 as compared to control.

In addition to the potential protein coding gene targets of SMAR1, a number of microRNAs were also predicted. As previously demonstrated, one of the microRNAs miR-373, showed p53-independent regulation by SMAR1. miR-371-373 has been shown to play a central role in tumor progression as well as maintenance of stem cell pluripotency^16^,^29^. Hence, we sought to elucidate the role of SMAR1 in the regulation of miR-371-373 in breast cancer tumorigenesis and metastasis. The ChIP-seq analysis predicted the binding of SMAR1 to a region upstream of miR-371, which is the first miRNA of the miR-371-373 cluster and the peak was only observed in HCT116 p53^−/−^ cells while it was absent in HCT116 p53^+/+^ cells (Fig. 4A). The MAR-Wiz (http://genomecluster.secs.oakland.edu/MarWiz/) analysis performed in this region showed a significant MAR potential, further projecting the probability of MAR-binding proteins like SMAR1 to bind to this sequence (Fig. 4B). Next, we performed chromatin immunoprecipitation using α-SMAR1 antibody and the eluted DNA was amplified using primers spanning this predicted SMAR1 binding region. The ChIP-PCR showed specific amplification in the immune pulled fraction, thus confirming the occupancy of SMAR1 at the miR-371-373 locus and validating the ChIP-seq data (Fig. 4C).

Taking into consideration the role of this miRNA cluster in cancer progression and metastasis, we were interested in understanding the role of SMAR1 in the transcriptional control of the microRNA cluster 371-373 in different breast cancer cell lines. Therefore, we chose different grades of breast cancer cell lines for further experimentation. HEK293 was taken as a representative of normal, non-cancerous cells; MCF-7 represented the non-metastatic breast cancer, while MDA-MB-231 and T47D were representative of metastatic breast cancer. We were then interested in determining the presence of other activator/repressor factors bound in association with SMAR1. It was found that SMAR1 was significantly enriched at the miR-371-373 promoter region in both HEK293 and MCF-7 cell lines. The binding of SMAR1 in these cell lines was associated with the recruitment of co-repressor molecules HDAC1/mSin3A, while the decreased occupancy of SMAR1 in MDA-MB-231 and T47D cell lines revealed a subsequent decrease in the enrichment of these co-repressor factors as well. Interestingly, the repressive histone mark H3K9me3 that was abundant in HEK293 and MCF-7 cells, was also found to be reduced at the miR-371 promoter in metastatic cell lines MDA-MB-231 and T47D (Fig. 4D). On the other hand, presence of RNA polymerase II and the activation histone mark H3K9Ac was enriched at this region in the metastatic breast cancer lines as compared to the non-metastatic ones (Fig. 4E,F). These data indicated that SMAR1 recruits co-repressor molecules HDAC1/mSin3A resulting in local chromatin condensation in this region. Absence of SMAR1 at the miR-371 promoter resulted in recruitment of activation marks in this region. We also confirmed SMAR1 binding upstream of the miR-371-373 cluster by electrophoretic mobility shift assay (EMSA). These assays were performed using the PCR amplified miR-371-373 upstream region as the template, against increasing concentrations of purified, recombinant SMAR1 protein. The results showed a specific binding of SMAR1 to the template DNA, resulting in a shift in the mobility of the complex as compared to the free probe. Further, a dose dependent increase was observed in the intensity of the SMAR1-template complex (Fig. 4G). This result depicted the specificity of SMAR1 binding to the miR-371-373 promoter.

To strengthen this observation, luciferase assays were performed using pluc371 and pluc371ΔMAR constructs (Fig. 4H). We observed that upon ectopic expression of SMAR1 in presence of the pluc371 vector there was decrease in luciferase activity, while SMAR1 knockdown increased the luciferase activity of the pluc371vector. This suggested and reconfirmed the recruitment of SMAR1 at the miR-371 promoter sequence. Additionally, there was negligible change in luciferase activities of the pluc371ΔMAR construct upon SMAR1 overexpression or knockdown (Fig. 4I). Thus, we concluded that there is specific interaction and recruitment of SMAR1 to the promoter of miR-371-373 cluster. This finding further led us to speculate the possible role of SMAR1 in the regulation of this cluster.

### SMAR1 negatively regulates the transcription of miR-371-373

**Figure 5:**
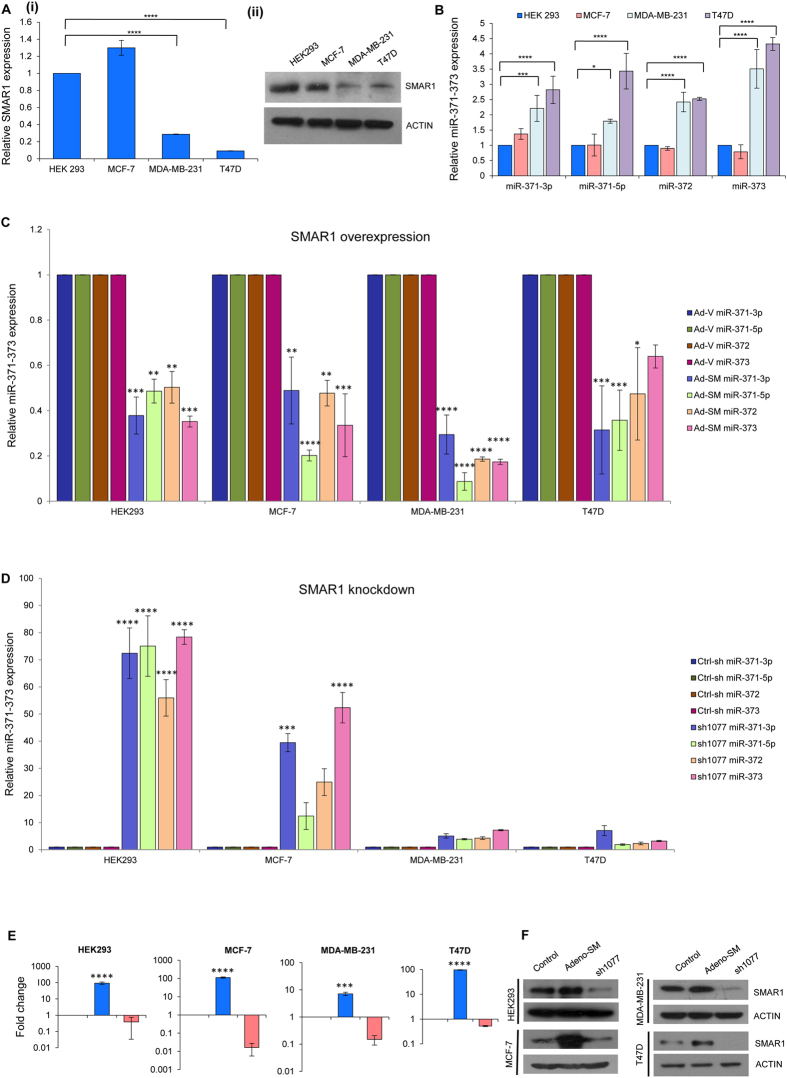
SMAR1 negatively regulates the transcription of the miR-371-373 cluster. (**A**) (i) Endogenous transcript and (ii) protein levels of SMAR1 detected in the four cell lines HEK293, MCF-7, MDA-MB-231 and T47D by quantitative real time PCR and western blot, respectively. (**B**) Quantitative PCR analysis to detect endogenous miR-371-373 transcript levels in these four cell lines. (**C**) Reduced levels of miR-371-373 transcripts upon ectopic expression of SMAR1 using Adeno-SM as determined by quantitative real time PCR in all four cell lines. Empty adenoviral vector (Ad-V) was used as control. (**D**) Upregulation of miR-371-373 transcript levels upon SMAR1 knockdown using sh1077 construct observed in four cell lines by quantitative real time PCR. shRNA construct was used as control. (**E**) Ectopic SMAR1 expression and knockdown was confirmed at transcript levels in HEK293, MCF-7, MDA-MB-231 and T47D by quantitative real time PCR. (**F**) SMAR1 overexpression and knockdown was confirmed at protein levels in HEK293, MCF-7, MDA-MB-231 and T47D by western blot assay. All the western blots are cropped representation of the original blots shown in Supplementary Fig. S8. All the gels are run under same experimental conditions.

As the cell lines HEK293, MCF-7, MDA-MB-231 and T47D have an inherent difference in their metastasis potential, we wanted to estimate the endogenous levels of SMAR1 and miR-371-373 transcripts in these lines. We observed that endogenous SMAR1 transcript and protein levels significantly decreased in the metastatic lines as compared to the non-metastatic ones, suggesting a negative correlation (Fig. 5A (i) and (ii)). The quantitative PCR analysis of the miR-371-373 transcripts indicated that the levels of miR-371-373 significantly increase with the metastatic ability of the breast cancer cell line (Fig. 5B). Thus, HEK293 which represented the normal cells showed the least expression of these miR transcripts endogenously and the maximum SMAR1 expression. On the other hand, the metastatic lines MDA-MB-231 and T47D showed the maximum expression of the miR-371-373 transcripts, with T47D showing the highest expression. We next wanted to determine the effect of SMAR1 on the transcription of the microRNAs in this cluster. It was observed that the transcript levels of miR-371-373 cluster were drastically reduced upon ectopic expression of SMAR1 in all the four cell lines (Fig. 5C). The knock down of SMAR1 was found to reverse this effect resulting in increased levels of these transcripts (Fig. 5D). Overexpression and knock down of SMAR1 was confirmed by quantitative real time PCR as well as immunoblot assays (Fig. 5E,F). Thus these results together indicate that SMAR1 regulates the expression of miR-371-373, transcriptionally.

### SMAR1 inhibits cancer cell migration and invasion by regulating miR-371-373 cluster

**Figure 6:**
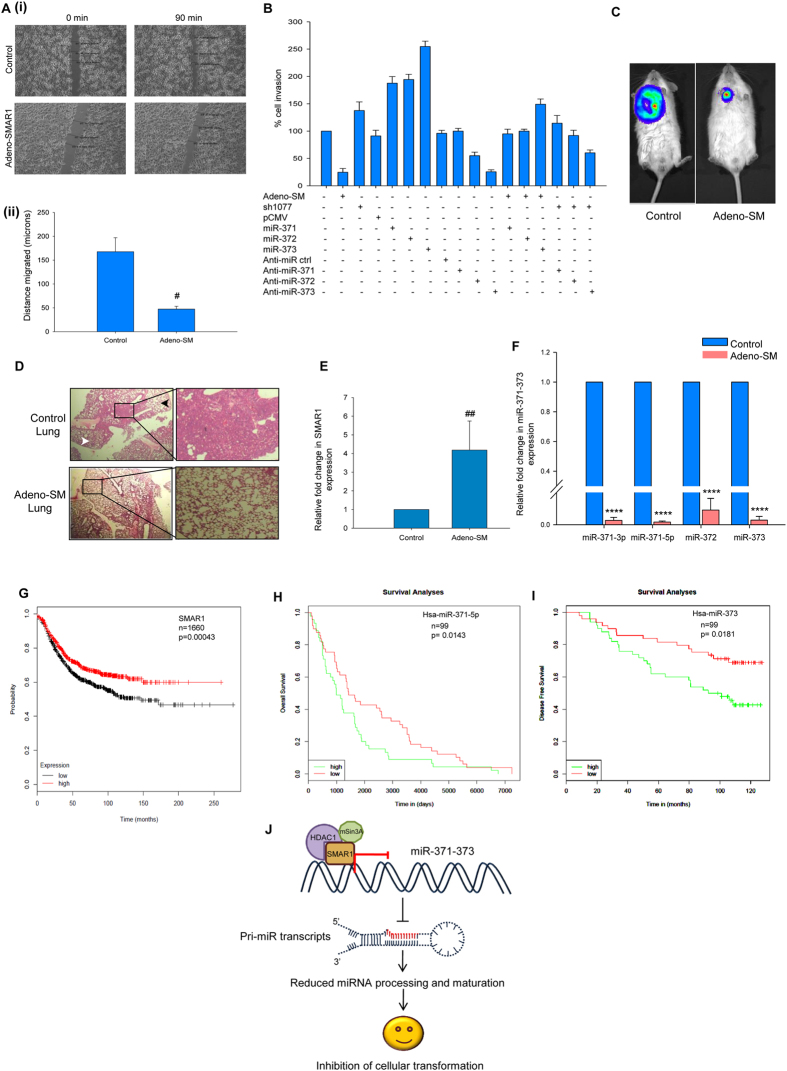
SMAR1 inhibits cancer cells *in vitro* and *in vivo* by regulating miR-371-373 cluster. (**A**) (i) *In vitro* wound healing assay in control and Adeno-SMAR1 transduced MDA-MB-231 cells showed reduced cell migration upon SMAR1 over expression. (ii) Graphical representation of the distance migrated (microns). (**B**) *In vitro* cell invasion assay performed in control MDA-MB-231 cells against different treatments of SMAR1 and miR-371-373 overexpression and/or knockdown alone and in combination. (**C**) *In vivo* imaging showing reduction in lung metastasis in mice injected with Adeno-SM transduced MDA-MB-231/Luc cells as compared to the mice injected with control MDA-MB-231/Luc cells. (**D**) Hematoxylin and Eosin staining of the lung sections of control mice showing alveolar collapse. Lung sections of Adeno-SM treated mice showing normal lung and alveolar architecture. (**E**) Quantitative real time PCR analysis showing increase in SMAR1 levels in the lung tissues of Adeno-SM treated mice. (**F**) Quantitative real time PCR analysis showing reduction in the miR-371-373 transcript levels in the lung tissues of Adeno-SM treated mice. (**G**) Kaplan-Meier relapse free survival analysis for SMAR1 in 1660 breast cancer patients. Lower expression levels of SMAR1 correlated with poor survival (p value = 0.00043). (**H**) Overall survival analysis for miR-371-5p in 99 breast cancer patients using MIRUMIR prediction tool. Higher expression levels corresponded to poor prognosis of patients (p value = 0.0143). (**I**) Disease free survival analysis for miR-373 in 99 breast carcinoma patients using MIRUMIR tool. Higher expression levels led to decreased survival of patients (p value = 0.0181). (**J**) Model depicting the inhibition of tumor progression and metastasis by SMAR1 mediated regulation of miR-371-373 cluster. Under normal scenario or in benign breast cancer, SMAR1 is present in physiologically normal levels in the cell thereby forming a repressor complex at the miR-371-373 promoter and repressing its expression. This allows the tumor suppressor mRNA targets of this cluster like LATS2 (Large Antigen Tumor Suppressor 2), CD44 to be translated preventing cellular transformation and maintaining homeostasis.

In order to establish the biological relevance of SMAR1 binding and repressing an important cluster of microRNAs reported to have a role in tumorigenesis and metastasis, we studied the role of SMAR1 in cell migration by wound healing experiment. MDA-MB-231 control and Adeno-SMAR1 transduced confluent cell monolayers were subjected to a wound and time lapse assay was performed to check for cell migration and wound healing. In comparison to the control cells, the SMAR1 overexpressed cells showed significantly retarded cell migration and wound healing (Fig. 6A (i) and (ii)). Next, we compared the cell invasion ability of control MDA-MB-231 cells against different treatments of SMAR1 and miR-371-373 overexpression and/or knockdown alone and in combination (Fig. 6B). It was observed that over expression of SMAR1 severely compromised the invasive capacity of these cells, while SMAR1 knockdown led to an increased invasiveness. The ectopic expression of miR-371, miR-372 and miR-373 enhanced cell invasion, and their respective antagomiRs inhibited it significantly. Over expression of miR-371, 372 and 373 along with Adeno-SMAR1 surpassed the inhibitory effects of SMAR1, making cells as invasive as the control. Similarly, the use of antagomiRs along with SMAR1 knockdown, compensated for the enhanced cell invasion potential, now making it comparable or less invasive than control MDA-MB-231 cells. Similarly, the *in vivo* tumorigenicity and metastasis experiments in SCID mice using control and Adeno-SMAR1 transduced MDA-MB-231/Luc cells indicated a perturbed metastases in the mice injected with SMAR1 overexpressed cells, while the control mice showed metastatic foci (Fig. 6C). Infiltration and metastasis of cells, accompanied by severe alveolar collapse is evident from the Hematoxylin and Eosin staining of the lung sections of control mice, while the Adeno-SMAR1 treated mice showed comparably normal lung and alveolar architecture (Fig. 6D). Quantitative real time PCR confirmed the increased levels of SMAR1 in the lung tissues of Adeno-SMAR1 treated mice (Fig. 6E). Parallely, we detected severely diminished levels of miR-371-373 transcripts in these lung tissues (Fig. 6F).

To extrapolate and confirm our findings, we utilized the Kaplan-Meier plotter for breast cancer model. The Kaplan Meier plotter is a tool capable of assessing the effect of 22,277 genes on survival using 10,188 cancer samples, of which 4,142 are breast cancer samples with a mean follow-up of 69 months^30^. We performed the survival analysis on 1660 relapse free survival (RFS) breast cancer patient data with respect to SMAR1 expression levels. The KM plot for SMAR1 established that lower expression levels of SMAR1 correlated with poor prognosis and survival (Fig. 6G). Next, we wanted to determine the expression pattern of miR-371-373 cluster in breast cancer patients and their survival. We exploited the MIRUMIR online survival analysis tool which utilizes multiple clinical data sets to derive information on microRNAs as biomarkers in cancers^31^,^32^. The survival analyses for miR-371-5p and miR-373 revealed that higher expression levels of these microRNAs in breast cancer directly associated with lesser patient disease free survival (n = 99) (Fig. 6H,I). However, no data was available for miR-371-3p and miR-372 with respect to overall patient survival. Therefore, we conclude that SMAR1 expression positively correlated with relapse free survival while miR-371 and miR-373 were predictors for poor prognosis. Taken together, SMAR1 curbs tumor formation, migration, invasion and metastasis through regulation of the miR-371-373 cluster (Fig. 6J).

## Discussion

Cancer is a micro-evolutionary process resulting from changes in the epigenome, loss of tight and controlled gene orchestration leading to, aberrant gene expression. Mutation or loss of tumor suppressor genes has been found to be one of the major causes of cancer initiation and progression. SMAR1 being a nuclear matrix protein, has the potential to regulate other vital processes in the cell as well. SMAR1 and p53 have been shown to mediate gene regulation in a competitive as well as co-ordinated fashion^6^,^26^. The outcome of gene expression also varies in either of these situations. With this aim, we proceeded to identify novel gene targets of SMAR1 in presence and absence of p53, by a high throughput ChIP-sequencing approach. Analysis suggested that SMAR1 can bind to a distinct set of genes in the presence and absence of p53. It was also found that p53 competes with SMAR1 for gene promoter occupancy as well as gene regulation altering the expression pattern of their common downstream gene targets (T cell receptor alpha variable 21 (TRAV21); leukocyte immunoglobulin-like receptor, subfamily B (LILRB); Y RNA (non-coding); U6 (non-coding small nuclear RNA); Clathrin Interactor 1 (CLINT1), and miR-373). For the same reason, we could observe significantly more number of SMAR1 bound peaks in the absence of p53 (HCT116 p53^−/−^ data set) than in the presence of p53. It is also notable to have identified two lncRNAs (RP11-271C24.2, RP11-419C23.1) as SMAR1 targets whose functions are still unexplored. The competitive binding of SMAR1 and p53 is appreciable in both these lncRNA genes and their gene regulation studies will provide new insights. p53 competing protein (p53CP), recently identified and characterized as p51/p63, has been shown to compete for binding at the p53 consensus sequence, thus inhibiting the transactivation potential of p53^33^,^34^. In another case, the interaction of the transcription factor Ets1 with p53 is indispensable for the CBP/p53 complex formation and stability and hence important for UV-responsive p53 transactivation in embryonic stem cells^35^. The overlapping of p53 (repressor) and hepatic nuclear factor 3 (HNF-3) (activator) binding sites in the alpha-fetoprotein (AFP) promoter results in competitive displacement of HNF-3 from this promoter^36^.

With respect to the distribution of SMAR1 peaks within the different gene components, it was found that SMAR1 binds within the gene body with higher affinity than to any other region of the gene. Studies from our lab suggest that proteins like SMAR1 that play a pivotal role in transcription-coupled alternate splicing of genes localise at the splice junctions in this manner. Hence, we speculate that SMAR1 has a major contribution in gene splicing and might be part of the splicing machinery of genes^37^. Also, the binding of proteins in the promoter and 5′ regulatory elements of genes are of utmost significance. Introns play an important role in regulating gene expression in eukaryotes. Introns in plants have been reported to enhance gene expression^38^. In the case of human ubiquitin C gene, the 5′UTR intron consists of a potent enhancer element that is critical for its transcription regulation^39^. Thus, existing literature in the field suggest potential role of SMAR1 in gene expression by binding to strong enhancer/regulatory elements of target genes. Binding sequence analysis of the SMAR1 target sequence reads identified T(C/G) repeat as the putative SMAR1 binding site in both the data sets irrespective of the status of p53. As ChIP-seq does not predict direct binding, the predicted SMAR1 binding repeat was validated by EMSA and ITC experiments. Presence of such repeats has been shown to have a functional role in binding of transcription regulators. CA repeats are thought to be conserved even in evolutionarily distant organisms and are likely to confer a unique conformation to the DNA^40^,^41^. Further, these sequences are observed to undergo CpG methylation, thereby playing a crucial role in gene repression. Such stretches of alternating purine/pyrimidine repeats confer a Z-DNA conformation *in vitro* and they have been implicated in both structure and regulation of eukaryotic chromatin^42^,^43^. We speculate that the presence of such repetitive doublets in the binding sequence probably confers a unique conformation to the chromatin in that region, which defines the specificity and affinity of SMAR1 binding at such sequences. This observation fits well owing to the established role of SMAR1 to be a transcriptional repressor of genes.

miR-371-373 is upregulated in higher grades of cancers, promoting metastasis and tumorigenesis. β-catenin/LEF1 has been reported to transactivate this microRNA-371-373 cluster that in turn regulates the Wnt/β-catenin-signaling pathway^44^. Previous studies from our lab have shown that levels of SMAR1 drastically decrease as the cancer progresses^5^. Taking these facts into consideration, we hypothesized that SMAR1 might regulate this miRNA cluster thereby promoting metastasis upon SMAR1 knock down/absence. Endogenous SMAR1 levels inversely correlate to the metastasis of the breast cancer lines, and thereby to all the microRNAs in the cluster miR-371-373. Thus, as the levels of SMAR1 in the non-metastatic cell line MCF-7 increased, the expression of all these miR transcripts, and thereby the metastatic property of the cells decreased. Further, ChIP-PCR confirmed the occupancy of SMAR1 at the miR-371-373 locus, validating the ChIP-seq data and the role of SMAR1 in its regulation. We identified the presence of repressor complex including HDAC1/mSin3A and H3K9me3 histone mark at the promoter alongwith SMAR1 in HEK293 and MCF-7 cell lines. In MDA-MB-231 and T47D cells, an activator complex including RNA pol II and H3K9Ac activator histone mark were detected. SMAR1 has been shown to promote ubiquitination and subsequent degradation of the histone acetyl transferase p300^45^. Therefore, we speculate that upon reduced endogenous levels of SMAR1, p300 might form a part of the activator complex inducing miR-371-373, as demonstrated by the presence of elevated H3K9Ac in such cells^46^. Real time PCR analysis of microRNAs upon SMAR1 overexpression and knock down reveals that SMAR1 negatively controls the transcription of all the four microRNAs, namely, miR-371-3p, miR-371-5p, miR-372 and miR-373. Thus, we conclude that SMAR1 exerts a transcriptional control on the expression of this microRNA cluster.

miR-371-373 has been implicated as oncomiRs in several types of cancers. Voorhoeve *et al*. in 2006^47^ reported that miR-371 and miR-372 act as oncogenes in testicular germ cell tumors. The over-expression of the miR-371 cluster alone has been demonstrated to overcome cell cycle arrest following introduction of RAS^V12^. Cellular transformation could be induced by the other two miRNAs in the cluster, miR-372 and miR-373 along with oncogenic RAS and WTp53. miR-373 has been reported to stimulate cell proliferation in human esophageal cancer by post-transcriptionally regulating large tumor suppressor, homolog 2 (LATS2)^48^. It has also been proposed to function as an oncogene in hepatocellular carcinoma via regulation of protein phosphatase 6^49^. An imprinted anti-miR-371-373 transcript, identified to act as an antisense regulator of onco-miR-372-3, functions as a tumor suppressor by cell growth arrest and apoptosis^50^. In pancreatic cancer patients, the increased expression levels of miR-371-5p were associated with significantly shorter survival and poor prognosis^51^. The overexpression of oncogenic miRNAs, miR-21, miR-10b, miR-155, miR-373 and miR-520c in breast cancers has been documented by many research groups. The mechanism by which miR-373 contributes to tumor progression and metastasis has been attributed to its ability of binding to the 3′UTR of CD44 mRNA and repressing its translation. The outcome of this regulation is upregulated levels of miR-373 and reduced levels of CD44 in clinical breast carcinoma samples. Similar findings have been reported in the case of enhanced invasion of prostate cancer by miR-373 and 520c as well^52^. In the light of these reports, we were interested in deciphering the effect of SMAR1 on breast cancer progression and metastasis by regulating this miR-371-373 cluster. Our experiments highlight the role of SMAR1 in inhibiting cancer cell migration, invasion, cancer progression and metastasis by regulating this critical cluster of microRNAs. These findings unravel a new mechanism by which SMAR1 executes its tumor suppressor function.

In summary, we have identified the potential of SMAR1 as a global gene regulator and chromatin modifier, orchestrating the expression of varied genes, including microRNAs. This study opens new horizons in the less explored field of transcription control of a cluster of microRNAs and its implications in breast cancer progression.

## Methods

### Cell lines and cell culture

HEK293, MCF-7, HCT116 p53^+/+^ and HCT116 p53^−/−^ cells were cultured in DMEM, T47D cells in RPMI, supplemented with 10% FBS and antibiotic and antimycotic (1X, Gibco) at 37 °C with 5% CO_2_. MDA-MB-231 and MDA-MB-231/Luc cells were cultured in Leibovitz’s L-15 medium with 10% FBS and antibiotic and antimycotic (1X) at 37 °C. The cultured cells were used for further experiments.

### Plasmids, transfections and transductions

The plasmids used for transfection experiment were prepared by Qiagen Midiprep kit. Transfection was carried out using Lipofectamine-2000 in OptiMEM without FBS. Cells were transfected with SMAR1 knockdown construct (sh1077) and harvested at 60 hours and protein extraction or RNA extraction was done. pCMV-miR-371, pCMV-miR-372 and pCMV-miR-373 constructs (Origene) were used for respective microRNA overexpressions as described above. Replication deficient recombinant SMAR1 adenovirus (Adeno-SM) was propagated in HEK293T cells and transduced in desired cells for 72 hours.

### Chromatin immunoprecipitation (ChIP)

Chromatin immunoprecipitation (ChIP) was done using HCT116 p53^+/+^ and HCT116 p53^−/−^ cells as described earlier^4^,^6^,^37^ using a chromatin immunoprecipitation (ChIP) assay kit (Upstate Biotechnology) following the manufacturer’s instructions. PCR was performed using different promoter specific primers.

### Chromatin immunoprecipitation-sequencing (ChIP-seq) and *in silico* analysis

Chromatin immunoprecipitation (ChIP) was carried out using anti-SMAR1 antibody (Catalog no. A300-279A, Bethyl Laboratories, Inc., USA) and normal IgG control from HCT116 p53^+/+^ and HCT116 p53^−/−^ cells as stated above. The eluted DNA fragments were subjected to single end sequencing using IlluminaGAIIx Analyzer as per manufacturer’s protocol. After adapter trimming using Cutadapt v1.2.1^53^ and confirming thread quality using FastQCv0.10.1 program (http://www.bioinformatics.babraham.ac.uk/projects/fastqc/), 14285728 and 25870976 good quality reads were obtained from HCT116 p53^+/+^ and HCT116 p53^−/−^ pulldown samples, respectively. These reads were then aligned to the human reference genome (hg19 Build) downloaded from UCSC genome browser^54^ using Bowtie2 v2.2.1 program^55^. The resultant sequence alignment map (sam) files were further converted to bam and bed format using SAMTools v0.1.18^56^. The bed files were used as the input to IGV2.3.26^57^ and MACS v1.4.2^58^ for visualization of alignment and peak calling, respectively. The obtained peaks were filtered on the basis of fold enrichment values. The nucleotide sequences corresponding to the peak coordinates were downloaded from UCSC genome browser and used as input to MEME-ChIP v4.10.1^59^ for motif analysis. The nearest downstream gene (NDG) function of PeakAnnotation option provided by PeakAnalyzer v1.4^60^ was used to annotate peaks. HCT116 p53^+/+^ and HCT116 p53^−/−^ data sets were then analyzed and compared for common and sample specific gene targets. The presence of the p53 consensus in the sequences corresponding to peaks was identified with the help of a custom Perl script. The gene ontology and pathway analysis were performed using DAVID Bioinformatics Resources 6.7^15^.

### Codes and softwares

All above softwares were downloaded as precompiled binaries and used on Linux platform. To handle sequencing data, finding consensus sequence and any for sort of automation, in-house custom Perl scripts were written and used. MAR-Wiz (http://genomecluster.secs.oakland.edu/MarWiz/) was used for determining MAR potential of the sequence of interest. The following sliding window parameters have been used: (i) Window width 1000; (ii) Slide distance 100; (iii) Cutoff threshold 0.60 and (iv) Run length 3.

### RNA extraction and quantitative real time PCR

Total cellular RNA was extracted using TRIzol reagent according to manufacturer’s (Sigma) protocol and first strand cDNA was synthesized from 2 μg RNA. For quantitative analysis of SMAR1 expression, Real Time PCR was performed by StepOne™ Real-Time PCR System (Applied Biosystems). In a 10 μl PCR reaction, cDNA was amplified using Power SYBR^®^ Green PCR Master Mix (Applied Biosystems), 50 pmol of forward and reverse primer mix. Confirmation of single product was determined by melt curve analysis. Comparative Ct method was utilised to quantify fold increase of specific mRNA(s) over control, where target was normalized to the endogenous GAPDH reference. The cycle number from logarithmic phase of the PCR curve where an increase in fluorescence was detected above background was recorded as the threshold Ct value. The ΔCt was determined by subtracting the Ct value of GAPDH from that of target. The fold increase over control = 2^−ΔΔCt^ where ΔΔCt = ΔCt_(control)_−ΔCt _(treatment)_.

### miRNA isolation and quantification

miRNA was isolated using *mir*Vana miRNA Isolation Kit (Ambion). In brief, the cells were lysed in a denaturing buffer, RNA was extracted using acid phenol: chloroform, and the samples brought to 25% (v/v) ethanol were passed through a glass-fiber filter. The filtrate was passed through a second glass-fiber filter, washed and eluted in a low ionic strength solution. Quantitative real time PCR was performed as described above. The relative abundance of the specific miRNAs was normalized to U6snRNA.

### Western blot analysis

SMAR1 and β-Actin protein levels were determined by immunoblotting. In brief, the proteins were extracted using protein extraction buffer (20 mM Tris-HCl pH-7.8, 1 mM EDTA, 1 mM PMSF, 0.1% (v/v) Triton X-100, PI cocktail) and then quantified using Bradford’s reagent (Bio-Rad). Equal amount of protein was loaded on 10% SDS-PAGE and samples were electrotransferred to PVDF membrane at 100 V constant voltage. The blots were then saturated with 5% (w/v) BSA/3% (w/v) non-fat dry milk and reacted with respective primary antibodies, then incubated with secondary antibodies tagged with horseradish peroxidase. Signals were detected by chemiluminescence using luminol as a substrate (Thermo Scientific).

### Electrophoretic mobility shift assay (EMSA)

Cy5-tagged (both at 5′ and 3′end) (TG)_10_ and (TC)_10_ oligos were used in combination with other competitive untagged oligos (AT)_10_, (GC)_10_, (TCTG)_5_ and alternatively with (TC)_10_ and (TG)_10_ oligos for repeat binding sequence confirmation. Purified recombinant SMAR1 protein with poly(dI-dC) [Poly(deoxyinosinic-deoxycytidylic), Sigma] (1 mg/ml) was incubated with the oligos alone or in different combinations, at room temperature for 1 hour in 50 mM Tris buffer (pH 7.5). The samples were then loaded on 8% native-PAGE pre-run at 100 V constant voltage at 4 °C for 1 hour and imaged in Typhoon FLA 9500 (GE Healthcare). PCR amplicon of miR-371-373 upstream MAR region was column purified and incubated with increasing concentrations of the purified recombinant SMAR1 protein and poly(dI-dC) for 1 hour at 37 °C. The sample was then loaded and resolved on a similar pre-run 10% native-PAGE at 4 °C. The gel was then stained with 0.5 μg/ml ethidium bromide, rinsed with water and visualized (Versadoc, Bio-Rad).

### Isothermal titration calorimetry (ITC)

The sample cell and Hamilton syringe were rinsed with water followed by buffer washes. The experimental parameters of the reaction carried out in MicroCal^TM^ iTC_200_ system (GE Healthcare) are: Total injections: 19; Cell temperature: 25°; Reference power (μcal/sec): 10; Initial delay (sec): 60; Stirring speed (rpm): 1000. Injection of 0.4 μl (duration 0.8 sec) was followed by 18 injections of 2.0 μl (duration 4 sec) with 150 seconds between injections. The purified recombinant SMAR1 protein (the macromolecule) was titrated against oligo sequence [(TG)_10_ or (TC)_10_] (the ligand). Data analysis was done using the MicroCal Analysis Launcher software.

### Luciferase reporter assay

To generate the SMAR1 binding site deletion mutant, PCR based site-directed mutagenesis was performed as described by Nassal and Rieger, 1990^61^. The ChIP-seq predicted SMAR1 peak in the promoter of miR-371-373 cluster was PCR amplified from genomic DNA using promoter-specific primers and cloned in pGL4.17[luc2/Neo] vector, referred to as pluc371 construct in this study. The MAR-deleted promoter was cloned using primers with compatible restriction site overhangs in pGL4.17 [luc2/Neo] vector, referred as pluc371ΔMAR construct. Cells were treated with control, Adeno-SM and sh1077 in combination with pluc371 or pluc371ΔMAR constructs and luciferase activities were measured after 60 hours of transfection by using the Dual Luciferase assay kit (Promega). pRL *Renilla* luciferase control reporter vector was used as internal control. The results were normalized to Renilla luciferase activity using Fluoroskan Ascent Luminometer (Lab Systems).

### Migration and invasion assays

A 10 μl pipette tip was used to generate an artificial wound onto confluent cell monolayers of control or Adeno-SM transduced MDA-MB-231 cells. The cell migration was then observed in serum containing medium upto 24 hours of the above mentioned treatments(s). Images were taken at 37 °C using Motorized IX-81 inverted microscope attached with DP70 CCD camera (Nikon). The distance migrated towards the wound was calculated after 90 minutes. MDA-MB-231 cells, alone or overexpressed with different pCMV-miR constructs or knocked down using antagomiRs in combination with SMAR1 over expression (Adeno-SM) or SMAR1 knockdown (sh1077 construct) were added to the upper chamber of the Boyden chamber (Corning). AntagomiRs used in the assay were anti-miR-371-3p, anti-miR-371-5p, anti-miR-372, anti-miR-373 and anti-miR-negative control (Ambion). The cells migrated to the reverse side of the upper chamber were fixed and stained with Crystal violet and counted under an inverted microscope (Nikon). Data are represented as the average of five fields per treatment well.

### *In vivo* tumorigenicity and imaging

All mice used in the animal experiments were bred at animal resource facility of National Centre for Cell Science (NCCS), Pune, India. All experiments were performed using standard protocols approved and monitored by institutional animal ethical committee of NCCS. All the methods were carried out in accordance with the approved guidelines of the animal ethical committee of NCCS. 10^6^ control and Adeno-SM transduced MDA-MB-231/Luc cells in sterile 1X PBS were injected into SCID mice via tail vein. The injected cells were then observed for their localization and/or metastasis by *in vivo* imaging. For this purpose, the mice were anesthetized, injected with luciferin (PerkinElmer) substrate (3 mg/20 g mice) and visualized under the *in vivo* imaging system (Xenogen). The mice were sacrificed and its vital organs were collected. Part of organs fixed in 10% (v/v) formalin was processed for Hematoxylin and Eosin staining (5 μm thickness) and imaged (Nikon). The remaining organ parts were used for total RNA and miRNA isolation.

### Survival curve analysis

Correlation of relapse free survival of breast cancer patients (n = 1660) with SMAR1 gene expression was analyzed using Kaplan-Meier Plotter with JetSet best probe set (http://kmplot.com/analysis/)^30^,^62^. Correlation of survival of breast cancer patients (n = 99) with hsa-miR-371-5p and hsa-miR-373 was performed using MIRUMIR online survival analysis tool (http://www.bioprofiling.de/MIRUMIR)^31^,^32^.

### Statistical analysis

All the experiments were performed at least thrice and the data are expressed as mean ± standard deviation. Statistical significance was calculated using either Student’s *t*-test or one-way ANOVA with a subsequent post hoc Tukey’s test for multiple comparisons. The significance values are defined as *p ≤ 0.05, **p ≤ 0.01, ***p ≤ 0.001, and ****p ≤ 0.0001 for ANOVA and ^#^p ≤ 0.05, ^##^p ≤ 0.005 for Student’s *t*-test. Densitometry analysis was done using the NIH ImageJ software (https://imagej.nih.gov/ij/).

## Additional Information

**How to cite this article**: Mathai, J. *et al*. SMAR1 binds to T(C/G) repeat and inhibits tumor progression by regulating miR-371-373 cluster. *Sci. Rep*. **6**, 33779; doi: 10.1038/srep33779 (2016).

## Supplementary Material

Supplementary Information

Supplementary Data 1
