# Supplementary material for: SMAR1 binds to T(C/G) repeat and inhibits tumor progression by regulating miR-371-373 cluster
Source: Sci Rep. 2016 Sep 27;6:33779. doi: 10.1038/srep33779 (PMC5037395; doi:10.1038/srep33779)
Supplement: Supplementary Information [file srep33779-s1.pdf]

SMAR1 binds to T(C/G) repeat and inhibits tumor progression by regulating miR-371-373 cluster

Jinumary Mathai[1], Smriti P.K. Mittal[2], Aftab Alam[1], Payal Ranade[1], Devraj Mogare[1], Sonal Patel[1], Smita Saxena[3], Suvankar Ghorai[1], Abhijeet P. Kulkarni[3] and Samit Chattopadhyay[1#*]

[1]Chromatin and Disease Biology Lab, National Centre for Cell Science, Savitribai Phule Pune University Campus, Ganeshkhind, Pune-411007, India

[2]Department of Zoology, Savitribai Phule Pune University Campus, Pune 411007, India

[3]Bioinformatics Centre, Savitribai Phule Pune University Campus, Pune 411007, India

[#]Present address: CSIR-Indian Institute of Chemical Biology, Raja S. C. Mullick Road, Jadavpur, Kolkata 700032, India

[*]To whom correspondence should be addressed: Dr. Samit Chattopadhyay, Tel: +91-33-24735368, Fax: +91-33-24735197; E-mail: samit@iicb.res.in

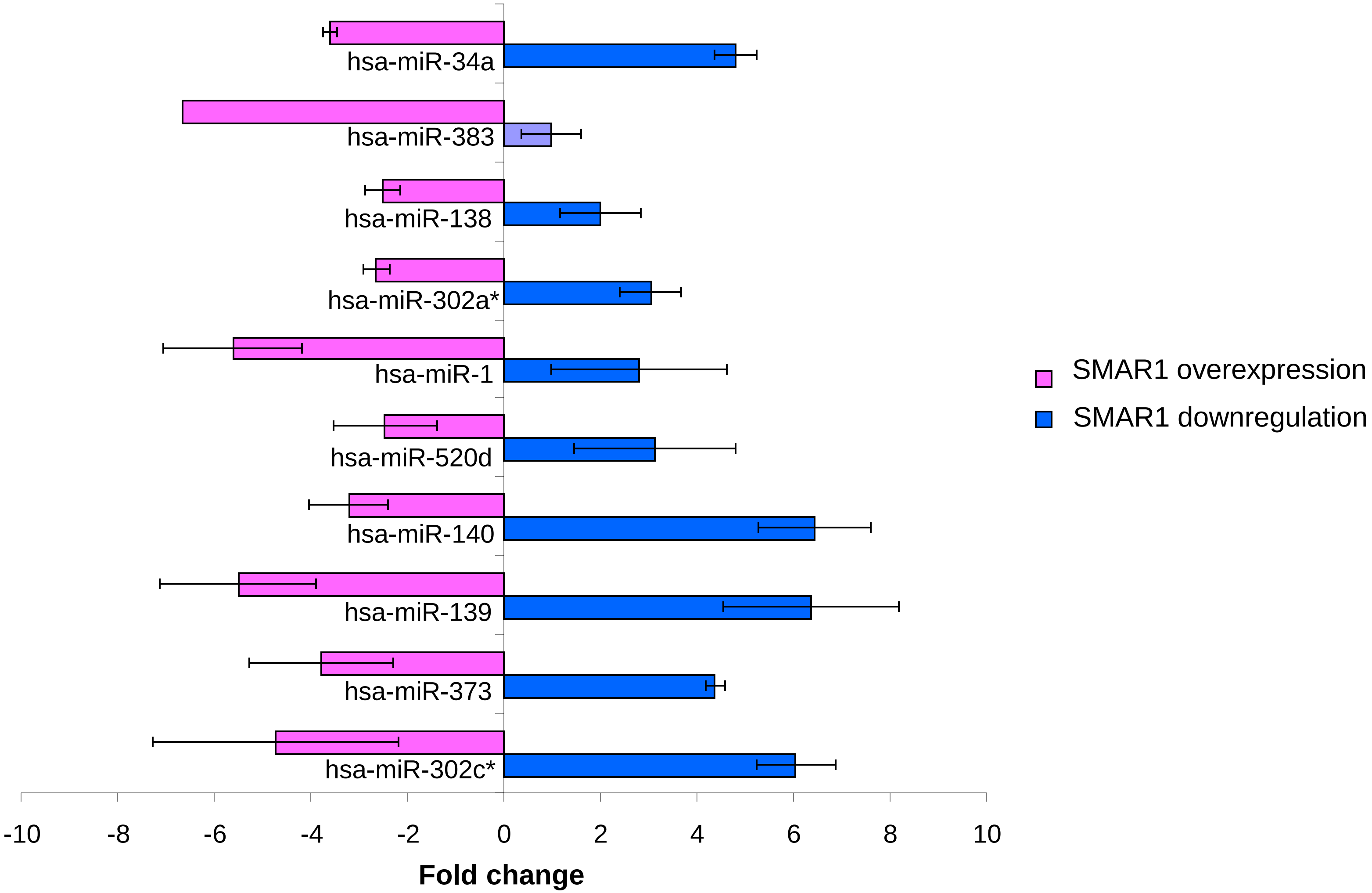

Supplementary figure S1: Representative data of miRNA microarray performed upon SMAR1 overexpression and knockdown in HCT116 $p53^{+/+}$.

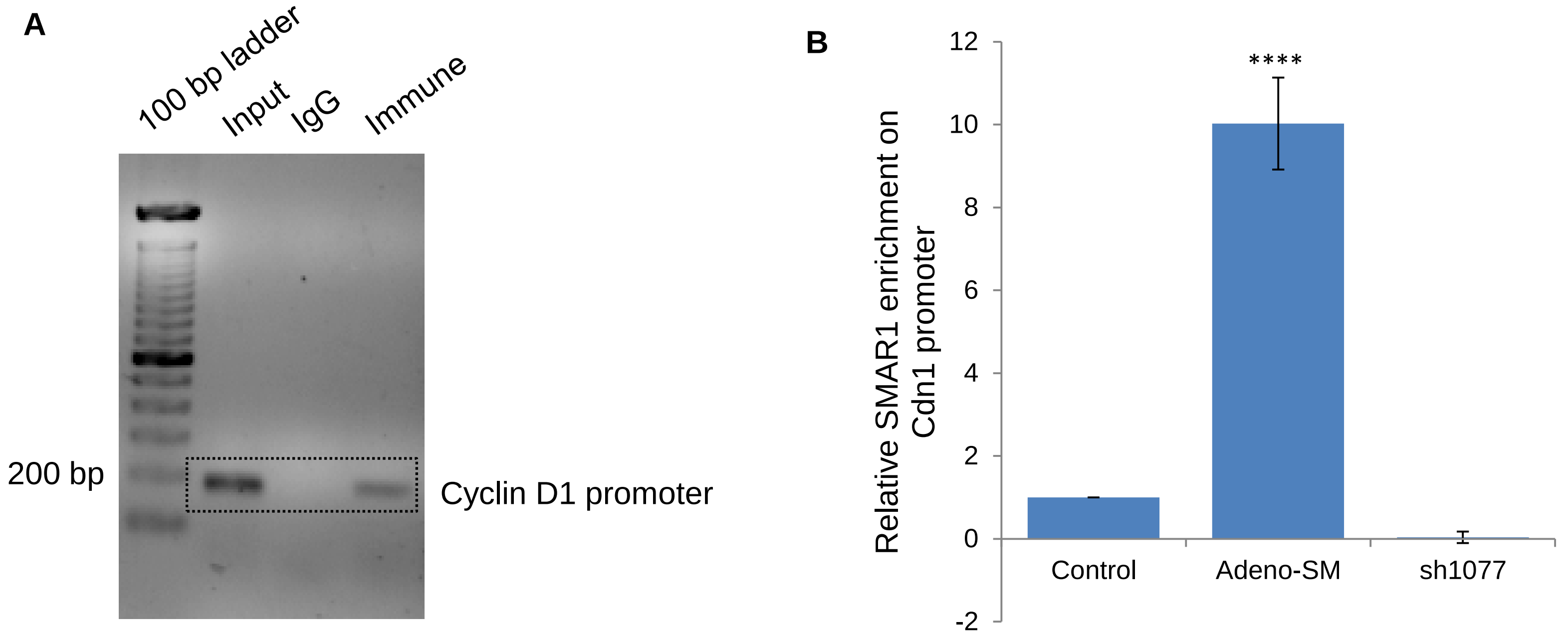

Supplementary figure S2: **A** Chromatin immunoprecipitation using anti-SMAR1 antibody (lane 3) to detect presence of SMAR1 on CyclinD1 promoter. Parallel immunoprecipitation with control rabbit IgG antibody is shown in lane 2. Lane 1 denotes input control.

B Relative enrichment of SMAR1 protein on Cyclin D1 promoter as detected by quantitative real time PCR. ChIP was carried out using anti-SMAR1 antibody upon SMAR1 overexpression and knockdown.

A

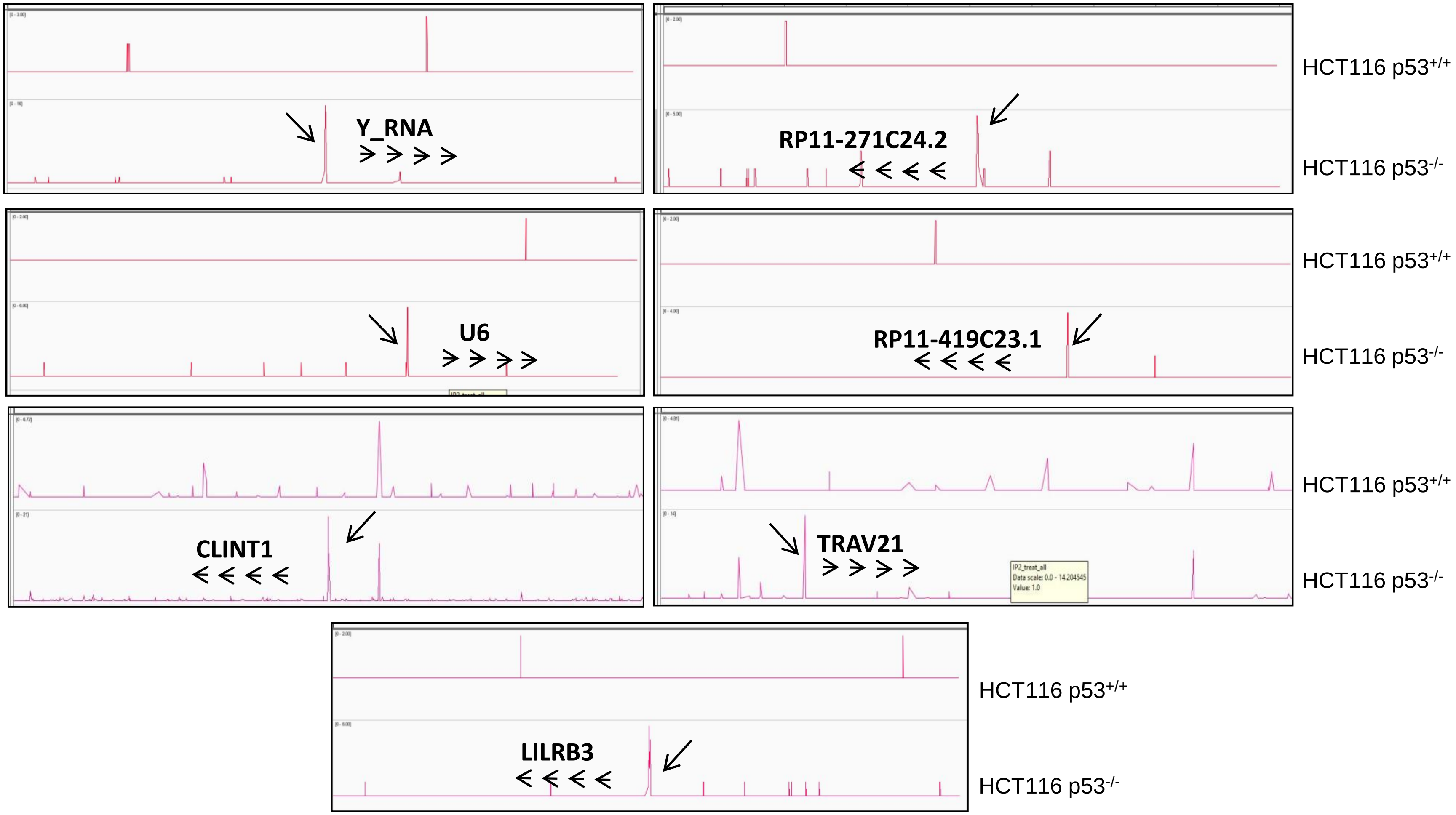

Supplementary figure S3A: Detailed visualization showing peak regions of the genes studied further in figure 3C and D. The peaks (indicated by arrows) were obtained using Integrative Genomics Viewer (IGV) in HCT116 $p53^{+/+}$ and HCT116 $p53^{-/-}$ cell lines. As expected the peak is observed in HCT 116 $p53^{-/-}$ only. Y-axis is Data values in bed graph file. X-axis represents genome co-ordinates.

B

GENE NAME	CORRESPONDING PEAK SEQUENCE
**TRAV21**	GTGTGAGAGAGAGAGAGAGAGAGGAGAGAGAGAGGAGAGAGAGAGAGAGAGAGAGAG<br>AAGAGAGAGAGAGAGAGAGAGAGAGAGACTTGTCTCAGATTGGCTGGGCCAACTCTGTGT<br>TTCCTGTAGAAATGTTTGAAC
**LILRB3**	GAGACAGACAGAGAGAGACGGAGAGACAGAGAGAGATTGAGACAGAGAGAGAGAGGGAG<br>AGGGAGACACACACACACACACACACGGAGAGAGACAGAGACAGA
**Y_RNA**	TCTAGAGCACACACACACACACACACACACACACGAGCATGTGCACATGCCCACCCAGTT
**U6**	CACACACACACACACACACACACACACACACACACACACACCTC
**CLINT1**	TGTAAGTAGGAGAGAGAGAGAGAGAGAGAGAGAGAGCGAAAGAGAGAGAAAGAGAGA
**miR373**	CATCTCCCTCTCTCTCTCTCTCTTCTCTCTCTCTCTCTCTCTCTCATATATATAT
**RP11-419C23.1**	TCCTGCTCTGGTGTGTGTGTGTGTGTGTGTGTGTGTGTGTGTGCGTGC
**RP11-271C24.2**	GATCACACACACACACACACACACACACACACACACACACACACACACGATT

Supplementary figure S3B: The sequence of peak region of the 8 genes used in the study and all the sequences are repeats of T(C/G).

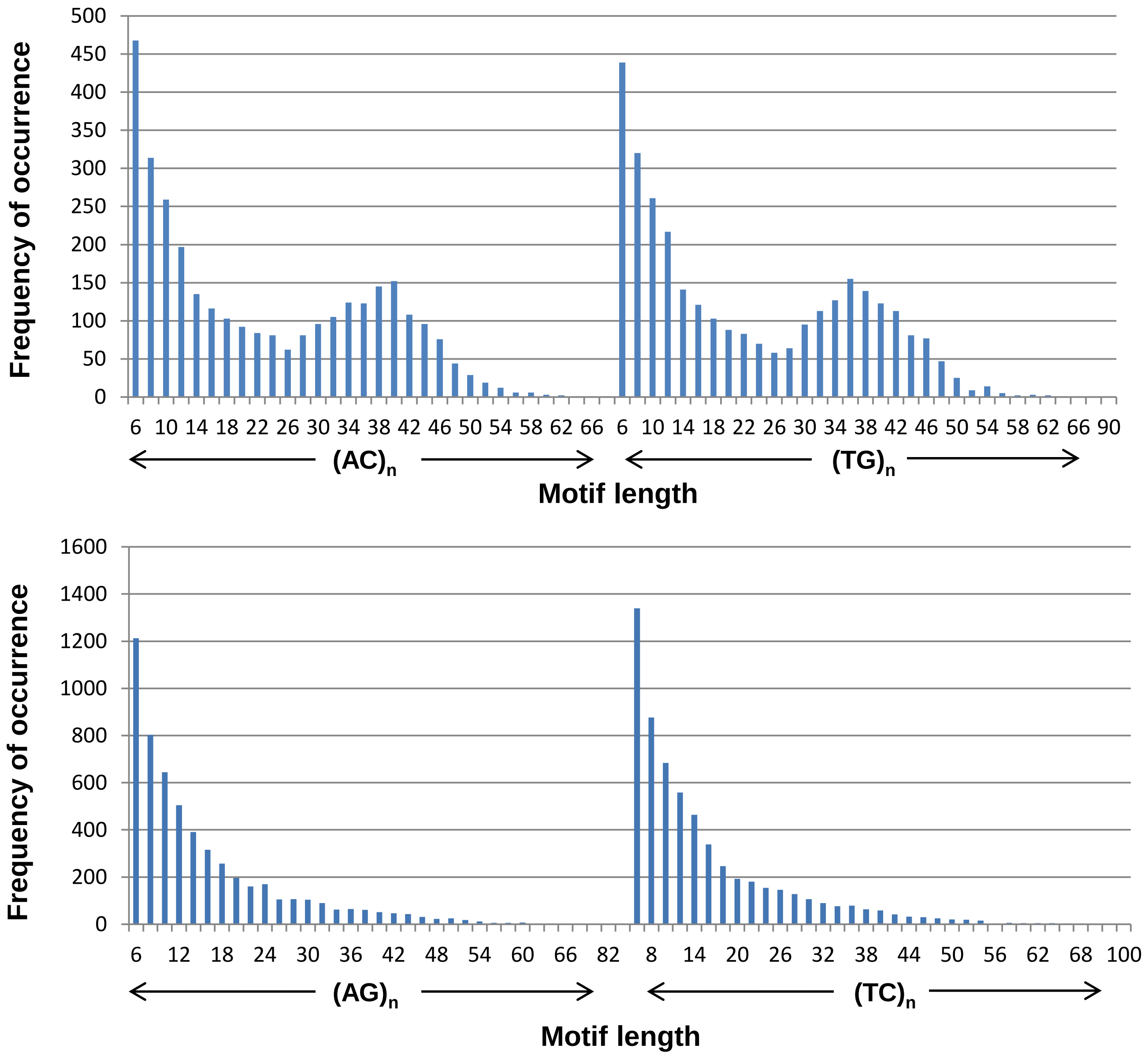

Supplementary figure S4: Histogram showing distribution of motif length and frequency of occurrence with motifs ranging in length from 6 to 100 bp.

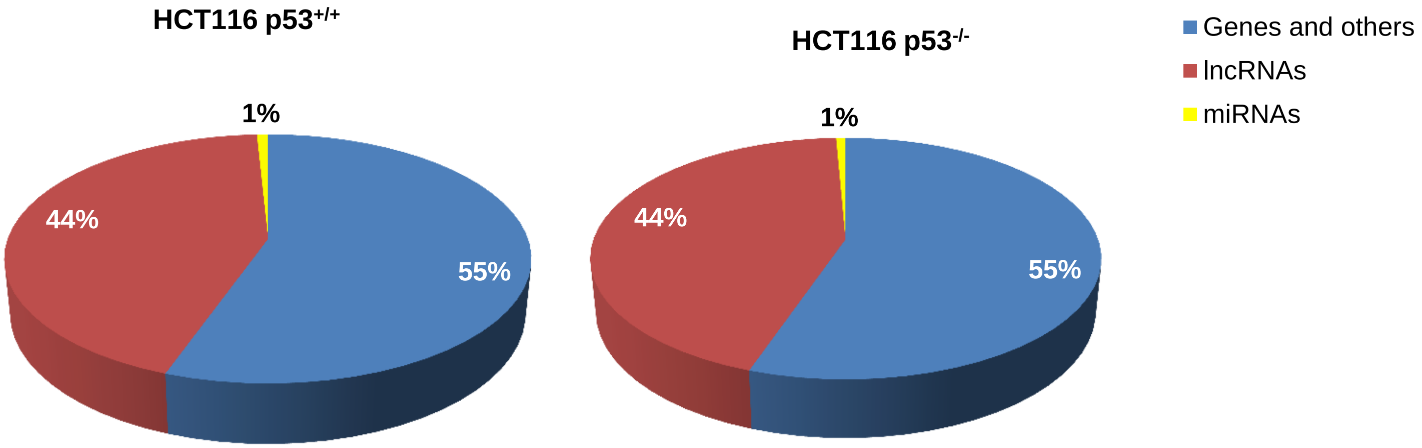

Supplementary figure S5: SMAR1 target genes categorized into protein coding genes, lncRNAs and miRNAs.

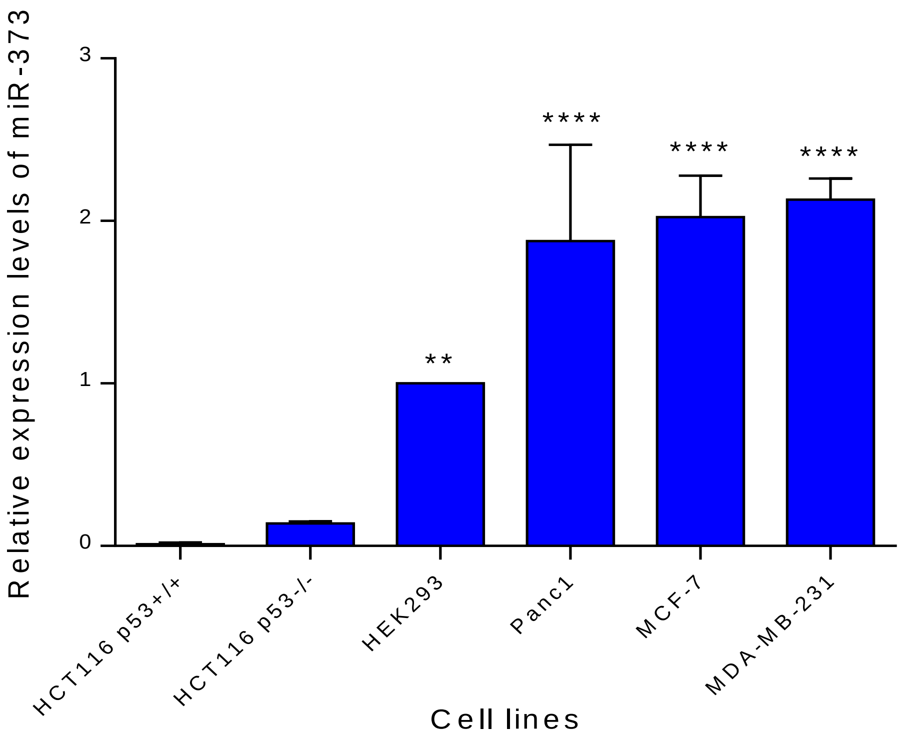

Supplementary figure S6: Quantitative PCR analysis to detect endogenous miR-373 transcript levels in different cell lines. HCT116 $p53^{+/+}$ was taken as reference. GAPDH was used as internal control.

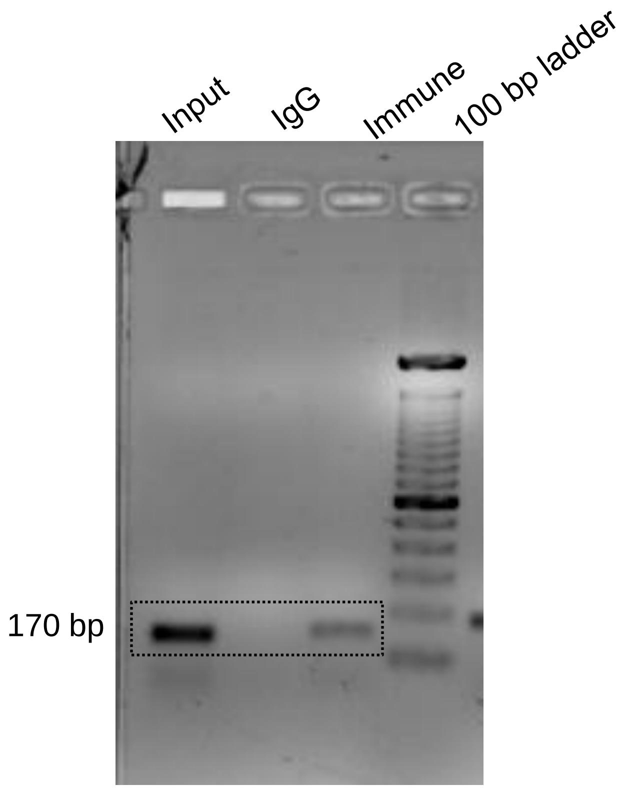

Supplementary figure S7: Original gel for figure 4C.

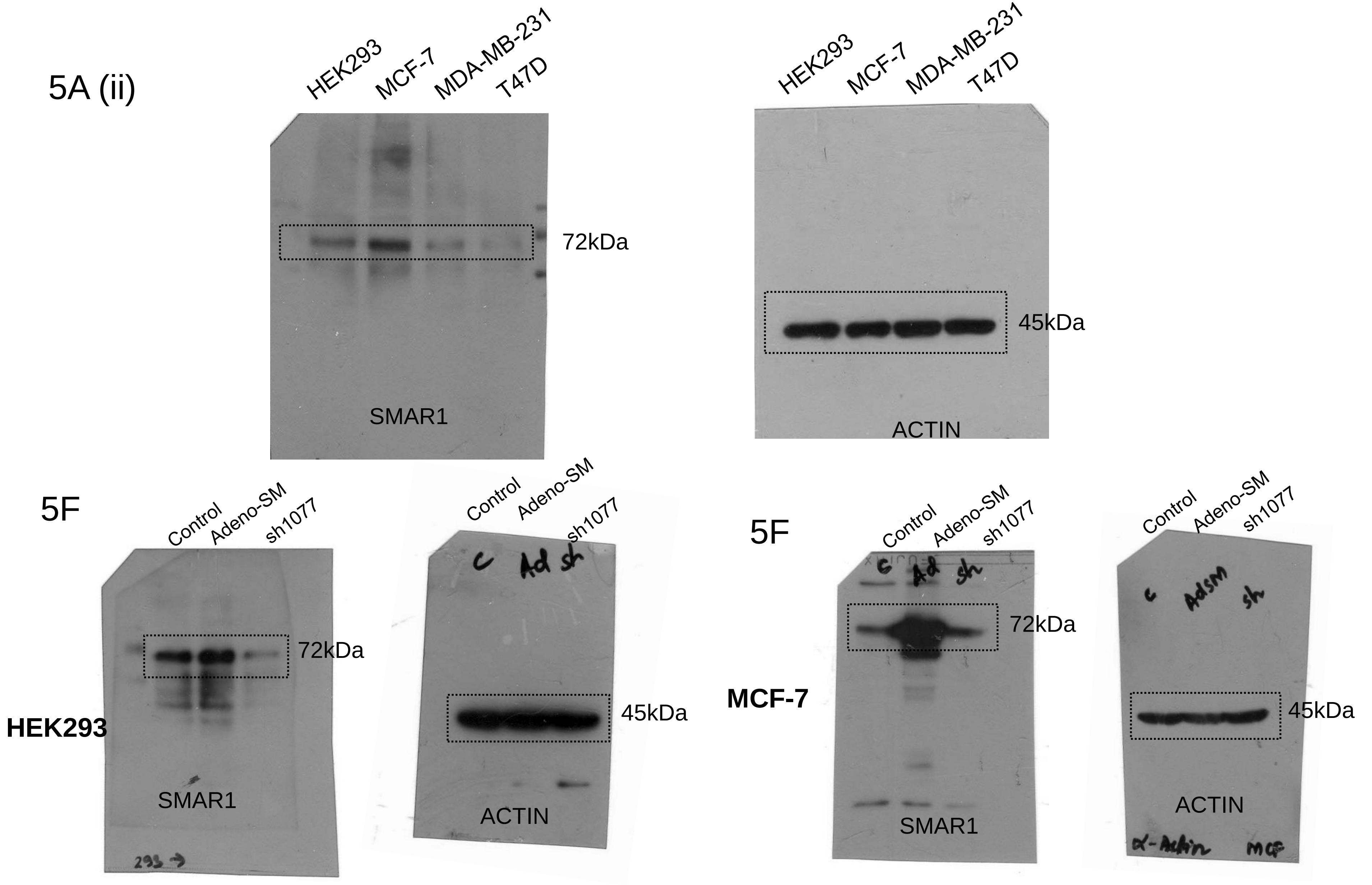

Supplementary figure S8: Original blots for figure 5.

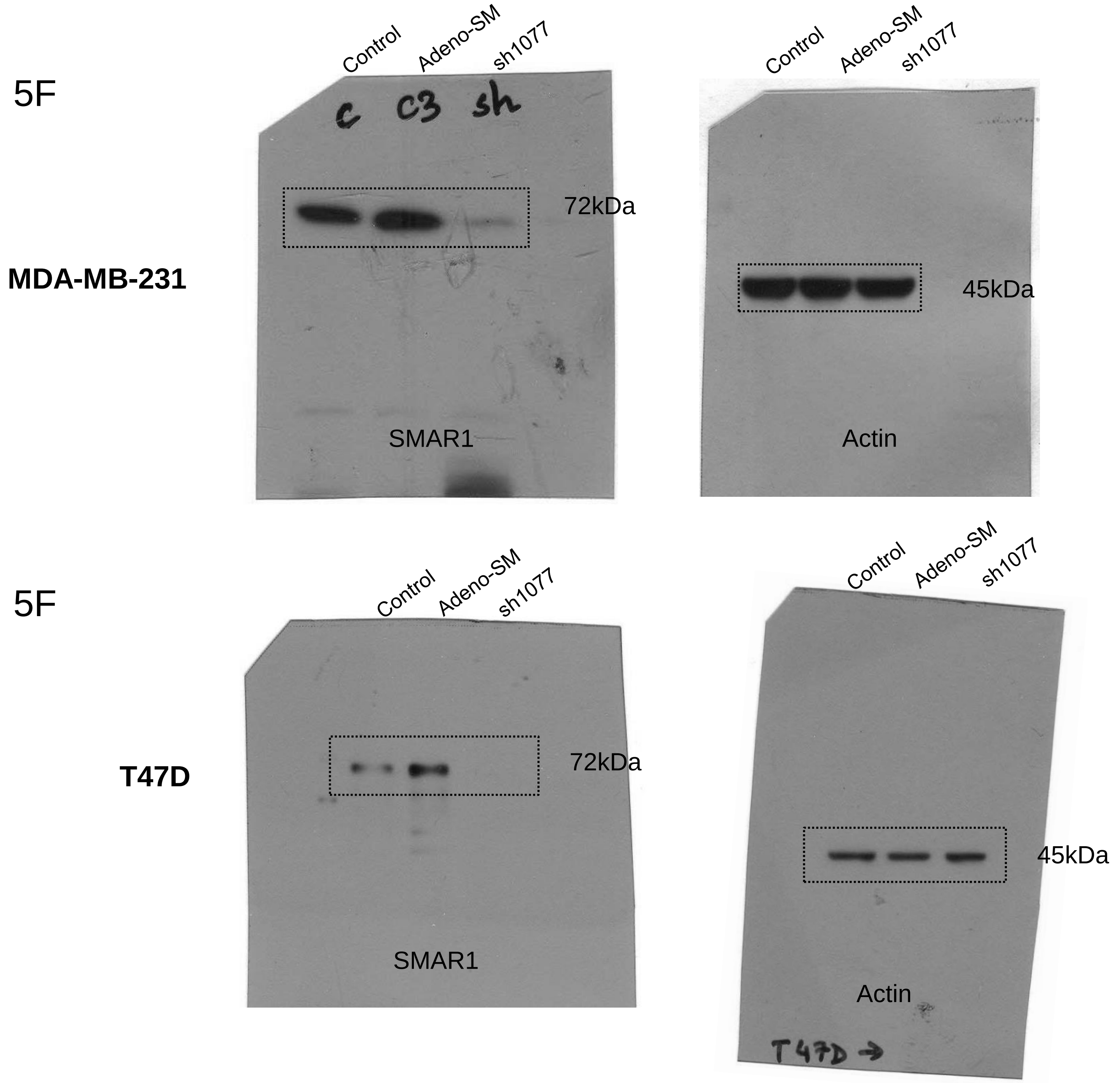

Supplementary figure S8: Original blots for figure 5.

Gene name	HCT116 $p53^{+/+}$		HCT116 $p53^{-/-}$
	p53 binding	SMAR1 binding	SMAR1 binding
RP11-271C24.2	+	-	+
RP11-419C23.1	+ + +	-	+ + +
TRAV21	+ +	-	+
LILRB	+ + +	-	+
Y RNA	+ +	-	+
U6	+ + +	-	+
CLINT1	+	-	+
miR-373	+ + +	-	+ + +

Supplementary Table S1: List of genes that harbor both SMAR1 and p53 motifs and used for further studies. '+' indicates presence and '-' indicates absence of binding.

**Alignments statistics**	**HCT116 p53$^{+/+}$**	**IgG1**	**HCT116 p53$^{-/-}$**	**IgG2**
Total reads	14285728	16604142	25870976	11732612
Aligned exactly 1 time	2653173 (18.57%)	4790245 (28.85%)	5838085 (22.57%)	4045656 (34.48%)
Aligned more than 1 time	1633133 (11.43%)	2626056 (15.82%)	3085070 (11.92%)	2196562 (18.72%)
Overall alignment rate	4286306 (30.00%)	7416301 (44.67%)	8923155 (34.49%)	6242218 (53.20%)

Supplementary Table S2: Bowtie statistics of ChIP-sequencing in HCT116 p53$^{+/+}$ and HCT116 p53$^{-/-}$ along with IgG controls.
